# Stage- and compartment-specific remodeling of autophagy and selective mitophagy in glaucoma: from aqueous outflow dysfunction to retinal ganglion cell neurodegeneration

**DOI:** 10.3389/fcell.2026.1842496

**Published:** 2026-06-04

**Authors:** Pai Zhou, Ying Deng, Yasha Zhou, Jing Lu, Qinghua Peng, Yijing Yang

**Affiliations:** 1 Hunan University of Chinese Medicine, Changsha, China; 2 Institute of Ophthalmology and Otolaryngology of Chinese Medicine, Changsha, China; 3 Centre for Innovation in Traditional Chinese Medicine Techniques for the Prevention and Treatment of Retinal Diseases and Visual Function Protection, Changsha, China

**Keywords:** autophagy, glaucoma, mitophagy, neurodegeneration, retinal ganglion cell, trabecular meshwork

## Abstract

**Background:**

Glaucoma is a leading cause of irreversible blindness and is increasingly understood as a chronic neurodegenerative disorder rather than a disease explained solely by elevated intraocular pressure (IOP). Although IOP lowering remains the cornerstone of treatment, many patients continue to progress despite apparently adequate pressure control, indicating that additional mechanisms shape retinal ganglion cell (RGC) vulnerability and disease course. Among these, autophagy and mitophagy have emerged as central regulators of cellular stress adaptation in both anterior and posterior ocular tissues.

**Main Body:**

This review argues that glaucoma can be more coherently interpreted through a stage- and compartment-specific framework of autophagy and selective mitophagy. In the conventional outflow pathway, autophagy contributes to mechanoadaptation, proteostasis, and extracellular matrix homeostasis, whereas chronic oxidative and biomechanical stress may impair lysosomal function and autophagic flux, thereby promoting outflow dysfunction and ocular hypertension. In the posterior segment, RGCs and their axons are highly dependent on autophagy for proteostasis and mitochondrial quality control because of their polarized morphology and substantial metabolic demand. Experimental work suggests that autophagy may be protective during early or acute stress but become insufficient, stalled, or maladaptive during chronic injury. Recent human stem cell and animal studies further implicate optineurin-linked autophagic-lysosomal dysfunction, AMPK–mTORC1 imbalance, and reduced PINK1/Parkin-associated mitophagy as mechanistic nodes linking mitochondrial stress to RGC degeneration. These observations support a model in which glaucoma progression reflects not simply more or less autophagy, but failure to maintain effective quality control across distinct ocular compartments and disease stages.

**Conclusion:**

A compartment-aware and time-resolved view of autophagy and mitophagy offers a more nuanced framework for glaucoma pathogenesis and therapy. Future progress will likely depend less on indiscriminate pathway modulation than on restoring selective, flux-competent quality control, particularly mitochondrial turnover, in the appropriate tissue and at the appropriate stage of disease.

## 1 Background

Glaucoma remains one of the most common causes of irreversible blindness worldwide. Its clinical importance lies not only in prevalence but in the fact that once retinal ganglion cells (RGCs) and their axons are lost, vision cannot be fully restored. A widely cited meta-analysis estimated that 76.0 million people aged 40–80 years were living with glaucoma in 2020, with this number projected to rise to 111.8 million by 2040 (Tham et al., 2014). This burden has reinforced the need to move beyond a narrow pressure-based view of the disease.

Elevated intraocular pressure (IOP) remains the best established and most tractable risk factor, and lowering IOP is still the only therapy proven to slow disease progression (Weinreb et al., 2014; Dervisevic et al., 2016). Yet pressure alone does not explain the full biological or clinical spectrum of glaucoma. Some individuals progress despite apparently adequate pressure control, whereas others tolerate elevated IOP for prolonged periods with relatively limited structural loss (Sayah et al., 2020). These observations have encouraged a broader conception of glaucoma as a chronic neurodegenerative disorder in which mechanical stress interacts with mitochondrial dysfunction, oxidative injury, inflammatory signaling, axonal transport failure, and declining cellular resilience (Dias et al., 2022; Kaur and Singh, 2023; Zhao et al., 2023; Zhang et al., 2026).

Within this expanded framework, autophagy has attracted growing attention. Autophagy is a lysosome-dependent degradative process that supports protein turnover, organelle recycling, energetic adaptation, and stress resistance. In long-lived, highly specialized cells, its importance is particularly evident. RGCs are metabolically demanding projection neurons with extensive dendritic arbors and long axons, and they therefore rely heavily on effective quality-control pathways to maintain homeostasis (Dias et al., 2022; Zhao et al., 2023). At the same time, cells in the conventional aqueous humor outflow pathway, especially trabecular meshwork (TM) cells, are exposed to chronic mechanical strain, oxidative stress, and extracellular matrix remodeling, conditions in which autophagy also appears central to adaptive capacity (Porter et al., 2013; Porter et al., 2015; Liton, 2016; Hirt and Liton, 2017; Shim and Liton, 2022). However, the role of autophagy in glaucoma has not been straightforward. Some studies support a protective function, especially in acute or early stress states, where autophagy induction appears to limit neuronal loss. Others suggest that chronic glaucomatous stress is associated with impaired lysosomal function, reduced autophagic flux, or maladaptive remodeling in both TM cells and neural tissues (Porter et al., 2013; Porter et al., 2015; Shim and Liton, 2022; Dixon et al., 2023). More recently, attention has shifted from bulk autophagy to selective mitochondrial turnover, or mitophagy. This is an important development because mitochondrial dysfunction is increasingly recognized as a central determinant of RGC vulnerability, and several recent studies indicate that reduced or ineffective mitophagy may contribute directly to glaucomatous neurodegeneration (Quinn et al., 2020; Jiménez-Loygorri et al., 2023; Chaphalkar et al., 2024).

Rather than asking whether autophagy is simply beneficial or harmful, the more useful question is where, when, and in what form autophagy becomes adaptive, insufficient, or maladaptive during disease progression.

This review therefore proposes a stage- and compartment-specific interpretation of autophagy and selective mitophagy in glaucoma. We argue that autophagy is not uniformly altered across the glaucomatous eye. Instead, it is remodeled across anatomically and functionally distinct compartments: the conventional outflow pathway, where autophagy influences IOP homeostasis; the retina and optic nerve, where autophagy supports proteostasis and mitochondrial quality control; and the glial-stromal microenvironment, where altered stress handling may amplify neuronal vulnerability (Liton, 2016; Alsemeh et al., 2023; Venkatesan and Bernstein, 2025). Within this framework, mitophagy deserves particular attention, not because it replaces broader autophagy biology, but because it offers a more selective and potentially more actionable lens through which to understand RGC degeneration (Quinn et al., 2020; Zhuang et al., 2022; Jiménez-Loygorri et al., 2023; Chaphalkar et al., 2024; Hu et al., 2024).

## 2 Conceptual framework: autophagy and selective mitophagy in glaucoma

### 2.1 Flux competence: distinguishing autophagy induction from successful degradation

Autophagy is not a single event but a sequence of coordinated processes that begins with cargo sequestration and ends with lysosomal degradation and recycling. In glaucoma, this distinction matters. An increase in autophagosomes or LC3-associated structures may reflect productive adaptation, but it may also indicate stalled degradation and unresolved cargo burden. The central issue is therefore not only whether autophagy is induced, but whether autophagic flux remains effective (Porter et al., 2013; Zhang et al., 2020).

This distinction is particularly relevant to chronic disease. In stressed TM cells, oxidative stress has been associated with lysosomal basification and reduced autophagic flux, suggesting that autophagy can be signaled while degradation remains incomplete (Liton et al., 2008; Porter et al., 2013). In chronic ocular hypertension models, changes in p62 and LC3-related markers have also been interpreted in a time-dependent manner, with evidence of early disruption and later partial compensation rather than a single stable state (Lee et al., 2021). These findings caution against reading autophagy markers in isolation.

### 2.2 Compartment specificity: different ocular tissues, different quality-control demands

A second organizing principle is compartment specificity. In the anterior segment, autophagy participates in adaptation to mechanical strain, oxidative stress, and extracellular matrix turnover within the conventional outflow pathway (Liton, 2016; Hirt and Liton, 2017; Shim et al., 2020; Tie et al., 2025). In the retina, by contrast, autophagy is closely tied to neuronal proteostasis, mitochondrial maintenance, and the metabolic demands of highly polarized RGCs (Zhu et al., 2017; Dias et al., 2022; Kaur and Singh, 2023). In the optic nerve, the problem is further complicated by axonal transport requirements and local energy dependence (VanderWall et al., 2020; Dias et al., 2022; Zhao et al., 2023). In glial cells, autophagy may shape trophic support, inflammatory tone, and non-cell-autonomous injury signaling (Jassim and Inman, 2019; Quillen et al., 2020; Gomes et al., 2022; Huang et al., 2024). These are related processes, but they are not interchangeable.

### 2.3 Stage dependence: adaptive, insufficient, and maladaptive autophagy across disease progression

A third principle is time dependence. Several experimental studies suggest that autophagy can be transiently protective during acute or early injury, yet become insufficient or dysregulated as stress persists (Deng et al., 2013; Russo et al., 2018; Lee et al., 2021). This temporal plasticity likely explains much of the apparent inconsistency in the literature. Early pathway activation may reflect adaptive stress handling; later accumulation of autophagic structures may instead mark failed clearance. The same pathway may therefore have different biological meaning at different points along the disease course (Russo et al., 2018; Lee et al., 2021; Dixon et al., 2023).

Mitophagy adds a further layer of selectivity to this framework. Bulk autophagy supports generalized turnover of cytoplasmic material, whereas mitophagy specifically removes damaged mitochondria. This emphasis on mitophagy does not imply that other forms of selective autophagy are unimportant in glaucoma. Mitophagy is highlighted primarily because mitochondrial quality control is closely linked to RGC bioenergetic demand, axonal maintenance, and glaucomatous neurodegeneration (Skeie et al., 2021). By contrast, in the conventional outflow pathway, selective or cargo-directed autophagy may be more relevant to ER stress, mutant or misfolded myocilin handling, lysosomal proteostasis, mechanotransduction, cytoskeletal adaptation, and extracellular matrix remodeling (Porter et al., 2013; Stothert et al., 2016; Shim et al., 2020). In glaucoma, this distinction is not trivial. RGCs are heavily dependent on mitochondrial function for ATP generation, calcium buffering, redox balance, and axonal maintenance (Irnaten and O’Brien, 2023; Liu et al., 2025). If mitochondrial quality control fails, the consequences include energy deficit, reactive oxygen species (ROS) amplification, and increased susceptibility to cell death signaling. Recent studies showing reduced mitophagy in rodent glaucoma models, together with neuroprotection conferred by PR-619 (a broad-spectrum deubiquitinating enzyme inhibitor), S3 (a small natural molecule reported to promote Parkin-mediated mitophagy), and mitochondrial serine hydroxymethyltransferase 2 (SHMT2, a mitochondrial one-carbon metabolism enzyme implicated in PINK1-related mitophagy regulation)-related interventions, suggest that mitochondrial turnover may be one of the more vulnerable and therapeutically relevant nodes in the disease (Zhuang et al., 2022; Chaphalkar et al., 2024; Hu et al., 2024; Liang et al., 2024).

**FIGURE 1:**
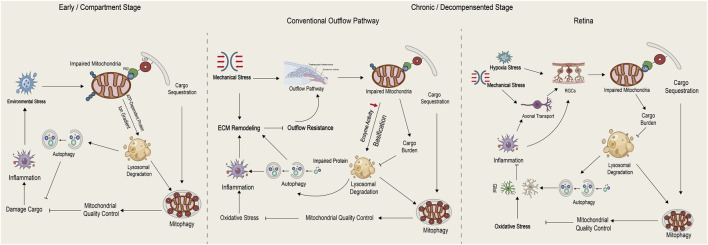
Stage- and compartment-specific remodeling of autophagy and selective mitophagy in glaucoma. Glaucoma is shaped by converging stressors, including elevated intraocular pressure, oxidative stress, mitochondrial injury, aging-associated lysosomal decline, inflammatory signaling, and genetic susceptibility. These inputs remodel the autophagy and selective mitochondrial quality control across distinct ocular compartments rather than producing a uniform response throughout the eye. In the conventional outflow pathway, altered autophagy may affect mechanoadaptation, extracellular matrix turnover, and aqueous humor outflow homeostasis. In the retina, retinal ganglion cells (RGCs) depend on effective autophagy and mitophagy for proteostasis and mitochondrial quality control. In the optic nerve and glial microenvironment, compartment-specific stress handling may further influence axonal integrity and neuroinflammatory tone. The schematic emphasizes a stage-dependent transition from early adaptive quality control to chronic insufficiency, stalled flux, or maladaptive remodeling, ultimately contributing to glaucoma progression.

Taken together, these observations support a working model in which glaucoma progression reflects a failure to maintain effective and compartment-appropriate quality control. Under early or limited stress, autophagy and mitophagy may buffer injury. Under sustained biomechanical, oxidative, inflammatory, or genetic stress, however, lysosomal competence may decline, cargo clearance may become inefficient, and mitochondrial quality control may fail (Jiménez-Loygorri et al., 2023; Chaphalkar et al., 2024; Hu et al., 2024). The result is not simply “too much” or “too little” autophagy, but a progressive uncoupling of stress sensing from stress resolution. A schematic overview of this stage- and compartment-specific framework of autophagy and selective mitophagy is shown in Figure 1.

### 2.4 Selective autophagy beyond mitophagy: compartment-specific cargo priorities

Aggrephagy refers to selective autophagic clearance of misfolded or aggregated proteins. This process is highly relevant to MYOC-associated glaucoma because mutant myocilin is retained in the endoplasmic reticulum, forms aggregation-prone species, induces ER stress, and compromises trabecular meshwork cell function. Experimental studies have shown that manipulating the ER–autophagy system or enhancing autophagic flux can promote mutant myocilin clearance and reduce ocular hypertension in MYOC-related models, supporting the view that aggregate and misfolded-protein handling is directly relevant to outflow-pathway proteostasis (Stothert et al., 2016; Kasetti et al., 2021). ER-phagy, the selective autophagic remodeling or degradation of ER subdomains, may also be relevant in this setting because mutant myocilin and chronic oxidative stress impose persistent ER proteostatic burden on trabecular meshwork cells. Although ER-phagy has not been fully characterized in glaucoma tissue, broader selective autophagy literature supports its role in resolving ER stress and maintaining secretory-cell proteostasis.

Lysophagy, the selective autophagic removal of damaged lysosomes, provides another potentially relevant mechanism. Oxidative stress can induce lysosomal basification and reduce autophagic flux in trabecular meshwork cells, while glaucomatous trabecular meshwork cells show broader autophagic dysregulation (Porter et al., 2013; Porter et al., 2015). Under such conditions, damaged or dysfunctional lysosomes may become a bottleneck for cargo degradation. Restoring lysosomal quality control, rather than simply inducing upstream autophagosome formation, may therefore be essential for preserving trabecular meshwork resilience. In parallel, autophagy is linked to mechanotransduction in outflow pathway cells. Trabecular meshwork and Schlemm’s canal cells are continuously exposed to cyclic mechanical strain and shear stress, and autophagy has been implicated in adaptation to these mechanical forces (Hirt and Liton, 2017; Shim et al., 2020; Shim et al., 2023). Through effects on cytoskeletal remodeling, focal adhesion turnover, extracellular matrix balance, and stress-induced senescence, selective and non-selective lysosomal degradation pathways may influence tissue stiffness, outflow resistance, and IOP homeostasis.

Thus, the compartmental emphasis differs across the glaucomatous eye. In RGCs and optic nerve axons, mitophagy may be especially important because mitochondrial integrity is central to ATP production, calcium buffering, redox balance, and axonal transport. In the conventional outflow pathway, aggrephagy, ER-phagy-related proteostasis, lysophagy, and lysosomal remodeling may be more directly linked to extracellular matrix remodeling, cytoskeletal adaptation, trabecular meshwork stiffness, and aqueous humor outflow. This distinction helps prevent overgeneralization of mitophagy and supports a broader, compartment-specific model of selective autophagy in glaucoma.

## 3 Compartment-specific remodeling from outflow tissues to the optic nerve

Because many glaucoma studies rely on whole-retina or single-compartment analyses of LC3, p62, or related markers, autophagy-related changes can be difficult to interpret without considering disease stage, tissue compartment, and flux competence (Porter et al., 2013; Lee et al., 2021). A compartment-based approach is therefore useful for reconciling apparently divergent findings across outflow tissues, RGCs, optic nerve axons, and glial cells. In reality, glaucoma unfolds across distinct yet interconnected compartments, each exposed to different forms of stress and each likely to engage autophagy in a different manner. A compartment-based approach helps explain why the literature has often yielded apparently divergent conclusions. Differences among studies may reflect not only technical variability, but also differences in disease stage, injury model, tissue compartment, and whether the measured markers reflect productive flux or stalled degradation.

In the conventional outflow pathway, autophagy appears integral to tissue homeostasis. Trabecular meshwork and Schlemm’s canal cells are continuously exposed to pulsatile pressure changes, oxidative stress, glucocorticoid influence, and extracellular matrix remodeling (Faralli et al., 2022; Shim and Liton, 2022). Under physiological conditions, autophagy contributes to adaptation under these demands by supporting cytoskeletal remodeling, focal adhesion turnover, mechanotransduction responses, lysosomal degradation of damaged proteins and organelles, and extracellular matrix balance (Liton, 2016; Mzyk et al., 2022). In this compartment, the most relevant cargo-selective processes may not be mitophagy alone. Aggrephagy and ER-phagy-related proteostasis are especially relevant to mutant or misfolded myocilin burden (Stothert et al., 2016; Kasetti et al., 2021), whereas lysophagy and lysosomal remodeling may become important when oxidative stress damages the lysosomal compartment itself (Porter et al., 2013; Porter et al., 2015). These pathways provide a mechanistic link between intracellular quality control, extracellular matrix turnover, cytoskeletal adaptation, trabecular meshwork stiffness, and aqueous humor outflow resistance. These processes are directly relevant to IOP homeostasis because trabecular meshwork contractility, tissue stiffness, extracellular matrix deposition, and Schlemm’s canal endothelial responses to shear stress all influence aqueous humor outflow resistance (Hirt and Liton, 2017; Shim et al., 2020). However, studies of oxidatively stressed trabecular meshwork cells suggest that chronic stress may not simply suppress autophagy, but impair its completion. Liton et al. showed altered lysosomal function in trabecular meshwork cells exposed to chronic oxidative stress, while Porter et al. further demonstrated lysosomal basification and reduced autophagic flux under oxidative stress. These findings are important because they shift the interpretation from “autophagy activation” to “autophagy inefficiency”: cells may initiate stress responses but fail to complete lysosomal degradation (Liton et al., 2008; Porter et al., 2013; 2015). In human glaucomatous trabecular meshwork cells, autophagic dysregulation has also been reported, supporting the possibility that impaired quality control contributes to outflow dysfunction and increased resistance (Porter et al., 2015; Shim and Liton, 2022). Nevertheless, these studies are largely cell-based or *ex vivo* in nature, and they do not by themselves prove that autophagy failure is the primary cause of ocular hypertension. A more cautious interpretation is that lysosomal–autophagic dysfunction may lower the adaptive capacity of outflow tissues under chronic biomechanical and oxidative stress.

In the retina, the biological meaning of autophagy appears more stage dependent. RGCs are highly specialized projection neurons with long axons, high energy demand, and limited regenerative reserve. These properties make them dependent on efficient proteostasis and mitochondrial quality control (Zhu et al., 2017; Tang et al., 2021; Dias et al., 2022). Several injury models show increased autophagy-related markers in RGCs after ocular hypertension, ischemia/reperfusion, optic nerve injury, or excitotoxic stress (Deng et al., 2013; Russo et al., 2018; Sano et al., 2019; Lee et al., 2021). However, increased LC3 or autophagosome accumulation does not necessarily indicate successful protection. In acute ischemia/reperfusion injury, rapamycin and fasting have been reported to sustain autophagy and promote RGC survival (Russo et al., 2018). This likely reflects a setting in which autophagy remains functionally adaptive and can still help clear damaged proteins or organelles. By contrast, in chronic ocular hypertension or spontaneous glaucoma models, persistent autophagy-related signaling may represent a different biological state. Lee et al. showed that the role of autophagy in RGC survival changed over time in a rat model of chronic ocular hypertension, suggesting that autophagy is not fixed as either protective or harmful (Lee et al., 2021). Dixon et al. further reported that autophagy deficiency protected against ocular hypertension and neurodegeneration in selected glaucoma models, an apparently paradoxical finding that may reflect maladaptive autophagy-related signaling, model-specific stress pathways, or reduced accumulation of toxic autophagic intermediates (Dixon et al., 2023). Therefore, the key distinction is not whether autophagy is “upregulated” or “downregulated,” but whether it remains flux-competent and beneficial at a given disease stage.

Mitophagy adds further specificity to this interpretation. Several recent studies suggest that glaucomatous injury is associated with inadequate mitochondrial turnover despite ongoing mitochondrial stress (Jassim et al., 2021a; Chaphalkar et al., 2024; Liu et al., 2025). Reduced mitophagy has been reported in rodent glaucoma models, and pharmacological enhancement of Parkin-mediated mitophagy by PR-619 or S3 has been shown to protect RGCs in experimental settings (Zhuang et al., 2022; Jiménez-Loygorri et al., 2023; Hu et al., 2024). These findings support the view that selective mitochondrial quality control is a vulnerable node in glaucoma. However, they should not be interpreted as evidence that all forms of autophagy activation are beneficial. PR-619 is a broad-spectrum deubiquitinating enzyme inhibitor, and S3 was tested in an excitotoxic retinal injury model rather than a fully chronic glaucoma model (Zhuang et al., 2022; Hu et al., 2024). Their protective effects therefore point to the therapeutic promise of mitophagy enhancement, but they also highlight the need to distinguish selective mitochondrial clearance from non-specific autophagy modulation.

The optic nerve introduces another layer of complexity. Axonal degeneration may not proceed in parallel with RGC Soma loss. Local energy failure, impaired axonal transport, mitochondrial accumulation, and altered organelle turnover may all shape autophagy differently in axons compared with cell bodies (Kitaoka et al., 2022; Liu et al., 2025). A pathway that appears partly adaptive in the Soma may already be insufficient in the axon. This spatial mismatch may help explain why global manipulation of autophagy produces inconsistent outcomes across studies (Lee et al., 2021; Dixon et al., 2023). It also supports the need to assess autophagy and mitophagy separately in RGC somata, axons, and optic nerve head glial compartments, rather than relying only on whole-retina marker analysis.

Finally, glaucoma cannot be understood as a purely neuron-centered disorder. Human stem cell-derived models carrying the glaucoma-associated OPTN (E50K) mutation show that autophagy-related dysfunction occurs not only in RGCs but also in astrocytes (VanderWall et al., 2020; Gomes et al., 2022; Huang et al., 2024). Co-culture studies further indicate that mutant astrocytes can modulate RGC neurodegenerative phenotypes, supporting a non-cell-autonomous component of glaucoma pathobiology (Jassim and Inman, 2019; Gomes et al., 2022; Huang et al., 2024). These findings broaden the framework from isolated RGC injury to a multicellular stress network. They also suggest that autophagic failure in 1 cell population may lower the resilience of another by altering trophic support, inflammatory tone, metabolic exchange, or extracellular homeostasis.

**FIGURE 2:**
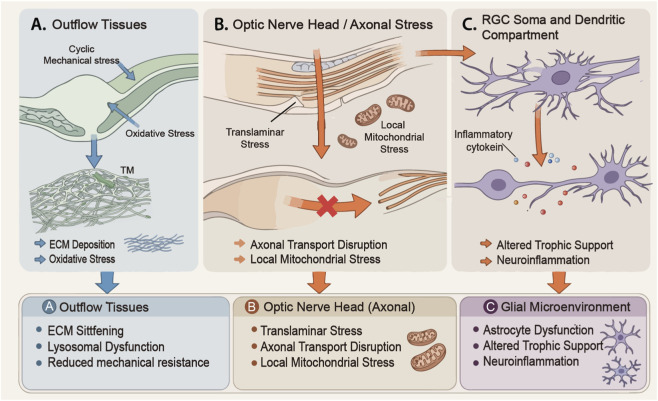
Compartment-specific mechanisms linking outflow dysfunction to retinal ganglion cell neurodegeneration. **(A)** Outflow Tissues. **(B)** Optic Nerve Head/ Axonal Stress. **(C)** RGC Soma and Dendritic Compartment. The glaucomatous eye can be viewed as a spatially connected system in which autophagy-related dysfunction develops across multiple compartments. In the conventional outflow pathway, chronic biomechanical and oxidative stress may impair lysosomal competence and autophagic flux in trabecular meshwork and Schlemm’s canal cells, contributing to extracellular matrix remodeling, increased outflow resistance, and ocular hypertension. Elevated pressure and biomechanical load are then transmitted to the optic nerve head, where axonal transport stress and local mitochondrial dysfunction may emerge. In retinal ganglion cells, chronic stress is associated with altered energy sensing, autophagic-lysosomal dysfunction, and impaired mitophagy, resulting in the accumulation of damaged mitochondria and progressive neuronal vulnerability. Glial dysfunction may further amplify this process through altered trophic support and inflammatory signaling. The schematic emphasizes that glaucoma-related neurodegeneration is shaped by interconnected, compartment-specific failures of cellular quality control rather than by a single uniform mechanism.

Taken together, the available evidence supports a spatially distributed and temporally dynamic model of quality-control failure. In the anterior segment, dysregulated autophagy may reduce the adaptive reserve of outflow tissues and contribute to ocular hypertension. In the retina, autophagy may be protective during early or acute stress but insufficient or maladaptive during chronic injury. In the optic nerve, axonal energy demand and transport stress may create a distinct vulnerability to defective mitochondrial quality control. In glial cells, altered autophagy may reshape the microenvironment that determines whether RGCs recover or degenerate. Representative studies are summarized in Table 1, with attention to the experimental system, ocular compartment, principal finding, interpretive implication, and major limitation. This format is intended to avoid treating heterogeneous models as equivalent evidence and to clarify why acute injury, chronic ocular hypertension, genetic stem-cell models, and pharmacological mitophagy studies may yield different conclusions. A compartment-based view linking anterior-segment dysfunction to posterior neurodegeneration is presented in Figure 2.

**TABLE 1:** Representative evidence supporting autophagy and mitophagy remodeling in glaucoma.

Study	Model/System	Compartment	Main finding	Interpreted implication	Major limitation
Liton et al. (2008)	Cultured porcine trabecular meshwork cells under chronic oxidative stress	Conventional outflow pathway/TM	Chronic oxidative stress altered lysosomal/autophagic activity in TM cells	Autophagy is involved in TM stress adaptation and may be disrupted early in outflow dysfunction	*In vitro* stress model; not glaucomatous tissue *in vivo*
Porter et al. (2013)	Oxidatively stressed TM cells	TM	Lysosomal basification and decreased autophagic flux were observed under oxidative stress	Chronic redox stress may impair autophagic completion rather than simply reduce pathway activation	Cell-based system; flux inference depends on experimental markers
Porter et al. (2015)	Human glaucomatous trabecular meshwork cells	TM	Glaucomatous TM cells showed autophagic dysregulation	Impaired autophagy may contribute to TM dysfunction and increased outflow resistance	Human cell findings do not directly establish causality
Russo et al. (2018)	Retinal ischemia/reperfusion injury	Retina/RGCs	Rapamycin and fasting sustained autophagy response and improved RGC survival	Early autophagy activation may be neuroprotective under acute stress	Acute injury model may not reflect chronic glaucoma biology
Lee et al. (2021)	Rat chronic ocular hypertension model	Retina/RGCs	The role of autophagy in RGC survival changed over time during ocular hypertension	Autophagy in glaucoma is stage dependent rather than uniformly beneficial or harmful	Dynamic flux remains difficult to define from marker-based data alone
Dixon et al. (2023)	Experimental and spontaneous glaucoma mouse models	Retina/optic nerve	Autophagy deficiency protected against ocular hypertension and neurodegeneration in selected models	Maladaptive autophagy-related signaling may contribute in some chronic settings	Findings appear context dependent and not easily generalized
VanderWall et al. (2020)	OPTN (E50K) human stem cell-derived retinal organoids	Retina/RGCs	Mutant RGCs showed neurodegenerative phenotypes with autophagy-related abnormalities	Glaucoma-associated genetic stress may directly perturb neuronal quality control	*In vitro* organoid model lacks full tissue complexity
Gomes et al. (2022)	OPTN (E50K) astrocyte–RGC co-culture system	Glial microenvironment/retina	Mutant astrocytes modulated RGC neurodegenerative phenotypes	Autophagy-related dysfunction may be non-cell-autonomous in glaucoma	Co-culture system does not fully capture *in vivo* glial complexity
Huang et al. (2024)	Isogenic OPTN (E50K) human stem cell-derived RGCs	Retina/RGCs	Autophagy disruption was associated with AMPK activation and reduced mTORC1 signaling	Impaired autophagic-lysosomal degradation may be linked to metabolic imbalance in glaucoma	Genetic model may represent a specific glaucoma subtype
Lin et al. (2023), Chaphalkar et al. (2024)	Rodent models of glaucoma	Retina/optic nerve	Reduced mitophagy was associated with glaucomatous neurodegeneration	Failure of mitophagy may be a key component of chronic glaucomatous stress	Association does not fully establish whether mitophagy loss is primary
Hu et al. (2024)	Experimental glaucoma	Retina/RGCs/optic nerve	PR-619 augmented Parkin-mediated mitophagy and protected RGCs	Enhancing selective mitophagy may offer neuroprotection in glaucoma	Pharmacological effects may extend beyond mitophagy alone
Zhuang et al. (2022)	Excitotoxic retinal injury	Retina/RGCs	S3 promoted Parkin-mediated mitophagy and protected RGCs	Selective mitochondrial quality control may be therapeutically actionable	Excitotoxic model is glaucoma-relevant but not glaucoma-specific

Representative studies suggest that autophagy- and mitophagy-related changes in glaucoma are compartment specific and model dependent. In the conventional outflow pathway, chronic stress is associated with lysosomal dysfunction and impaired autophagic flux. In the retina and optic nerve, both protective and maladaptive autophagy-related responses have been reported, while more recent work implicates impaired mitophagy as a potential contributor to retinal ganglion cell vulnerability.

## 4 Glial autophagy and non-cell-autonomous amplification of RGC vulnerability

Glaucoma is increasingly understood not only as an RGC-intrinsic degenerative process, but also as a multicellular disorder in which glial cells actively shape neuronal vulnerability. Astrocytes, Müller glia, and microglia are positioned to regulate extracellular homeostasis, metabolic support, inflammatory tone, synaptic and axonal stress responses, and debris clearance. Therefore, autophagy in these supporting cells may influence RGC survival in a non-cell-autonomous manner. This is particularly relevant because glial cells are among the earliest responders to ocular hypertension, optic nerve head stress, mitochondrial dysfunction, and tissue inflammation in glaucoma. Reviews of glial biology in glaucoma have emphasized that astrocytes, Müller cells, and microglia can shift from homeostatic support to maladaptive reactivity, thereby modifying the retinal and optic nerve microenvironment in which RGCs attempt to survive (García-Bermúdez et al., 2021).

Astrocytes provide one of the clearest examples of glia-mediated modulation of RGC phenotypes. In human pluripotent stem cell-derived models carrying the glaucoma-associated OPTN (E50K) mutation, astrocytes displayed disease-related abnormalities, including evidence of autophagy dysfunction, and altered their support of RGCs in co-culture (Gomes et al., 2022). Showed that OPTN (E50K) astrocytes released reduced levels of neurosupportive factors and modulated neurodegenerative phenotypes in OPTN (E50K) RGCs, supporting the concept that glaucoma-associated genetic stress can operate through glial as well as neuronal compartments (Gomes et al., 2022). This finding is important for the present framework because OPTN is not merely a glaucoma gene; it is also involved in selective autophagy and cargo handling. Thus, impaired autophagy in astrocytes may reduce their ability to maintain trophic support, buffer extracellular stress, and restrain inflammatory signaling, thereby lowering the threshold for RGC degeneration.

Microglia provide a second route through which glial quality-control failure may amplify neuronal injury. In glaucoma models, microglial activation has been associated with neuroinflammatory responses and RGC damage, and the pathway involving the P2X7 receptor (P2X7R) and NLRP3 (NLR family pyrin domain containing 3) pathway has been implicated in chronic ocular hypertension-related microglial activation and RGC injury (Zhang et al., 2019; Ishikawa et al., 2023). Mechanistically, defective autophagy or mitophagy may increase the persistence of damaged mitochondria, mitochondrial reactive oxygen species, and danger-associated molecular signals, which can contribute to inflammasome priming or activation. Although direct glaucoma-specific evidence linking glial mitophagy failure to NLRP3 activation remains incomplete, the convergence of mitochondrial stress, autophagy impairment, and microglial inflammasome signaling provides a plausible mechanism by which local quality-control failure becomes a tissue-level inflammatory amplifier. This interpretation also helps explain why mitochondrial dysfunction in glaucoma should not be viewed solely as an RGC-intrinsic energetic defect, but as a trigger for glia-neuron inflammatory crosstalk.

Müller glia further extend this non-cell-autonomous framework. As the principal retinal macroglia, Müller cells regulate glutamate handling, potassium buffering, antioxidant support, metabolic exchange, and inflammatory signaling. Experimental work has shown that interactions between Müller cells and microglia can aggravate retinal inflammatory responses, indicating that macroglia–microglia crosstalk may influence neuronal stress propagation (Hu et al., 2021). More recent studies also suggest that Müller glia can support RGC survival through growth-factor-mediated and homeostatic mechanisms, reinforcing the idea that disruption of Müller cell stress handling may indirectly compromise RGC resilience (Cheng et al., 2025). In this context, glial autophagy should be considered a regulatory process that helps maintain retinal immune privilege and metabolic stability rather than a housekeeping pathway alone.

Together, these observations support a broader model in which dysfunction of autophagy and selective mitochondrial quality control is distributed across both neuronal and non-neuronal compartments. RGC degeneration may therefore arise not only from cell-autonomous failure of mitochondrial quality control, but also from astrocytic loss of trophic support, microglial inflammatory activation, and Müller glial disruption of retinal homeostasis. Therapeutically, this argues against a purely RGC-centered strategy. Durable neuroprotection may require restoring quality-control competence in the glial microenvironment while preserving RGC-intrinsic mitophagy and axonal resilience.

## 5 Mitophagy failure, mitochondrial danger signals, and NLRP3 inflammasome activation

Mitophagy failure provides a plausible mechanistic link between mitochondrial dysfunction, lysosomal stress, and chronic neuroinflammation in glaucoma (Jassim et al., 2021b). Canonical mitophagy ultimately requires lysosomal degradation; therefore, mitochondrial tagging or mitophagosome formation alone cannot guarantee effective mitochondrial clearance. This distinction is important because lysosomal function itself may decline under oxidative stress, aging, and chronic glaucomatous injury (Porter et al., 2013; Yan et al., 2026). Lysosomal acidification, hydrolase activity, autophagosome–lysosome fusion, and recycling capacity all require adequate ATP supply, ion homeostasis, and intact lysosomal machinery (Kim et al., 2025; Kiraly et al., 2025). Mitochondrial dysfunction may therefore indirectly compromise lysosomal degradation by reducing cellular energy availability and increasing oxidative stress, whereas lysosomal impairment may in turn prevent efficient removal of damaged mitochondria (Boya et al., 2023; Kim et al., 2025). This reciprocal relationship can create a quality-control bottleneck in which damaged mitochondria accumulate despite upstream autophagy or mitophagy signaling.

The therapeutic implication is that mitophagy enhancement should be interpreted according to the state of downstream lysosomal competence (Wang Q. et al., 2026). In cells with preserved or partially impaired lysosomal function, promoting mitochondrial recognition and delivery to lysosomes may improve clearance of damaged mitochondria and reduce mitochondrial reactive oxygen species, mitochondrial DNA leakage, and other danger signals (Stavropoulos et al., 2023; Yang et al., 2024). In cells with severe lysosomal failure, however, upstream mitophagy induction alone may be ineffective or even increase undegraded cargo burden (Porter et al., 2013). Under those conditions, mitochondrial quality control may require combined approaches that restore lysosomal function while also limiting mitochondrial injury (Shim and Liton, 2022).

It is also important to recognize that mitochondrial quality control includes mechanisms beyond canonical lysosome-dependent mitophagy. Ubiquitin–proteasome-dependent degradation of damaged outer mitochondrial membrane proteins can occur upstream of, or parallel to, complete mitochondrial engulfment (Karbowski and Youle, 2011; Taylor and Rutter, 2011). Such mechanisms may reduce the burden of damaged mitochondrial components and help contain mitochondrial danger signaling even when lysosomal clearance is incomplete. Although the relevance of these lysosome-independent mitochondrial quality-control pathways remains underexplored in glaucoma, they provide an important conceptual addition to the therapeutic framework (Das et al., 2020). They suggest that future interventions should not be evaluated solely by whether they increase mitophagy markers, but by whether they restore functional mitochondrial homeostasis, reduce mitochondrial danger signaling, and preserve RGC and glial resilience.

Under physiological stress, damaged mitochondria are selectively recognized and removed mainly through mitophagy, thereby limiting excessive mitochondrial reactive oxygen species (mtROS), mitochondrial DNA (mtDNA) leakage, impaired calcium buffering, and release of mitochondrial danger-associated molecular patterns (Charmpilas et al., 2023). When this clearance process becomes inefficient, dysfunctional mitochondria are not merely passive remnants of cellular injury; they can become active sources of inflammatory signaling (Marchi et al., 2023). This concept is particularly relevant to glaucoma because mitochondrial abnormalities, oxidative stress, and glial inflammatory activation have all been implicated in glaucomatous neurodegeneration (Stavropoulos et al., 2023). A glaucoma-focused review by Jassim et al. emphasized that crosstalk between damaged mitochondria and inflammatory signaling may enhance pathology in glaucomatous neurodegeneration, supporting the view that mitochondrial stress can help convert local cellular injury into a broader neuroinflammatory response (Jassim et al., 2021b).

One important downstream pathway is the NLRP3 inflammasome. NLRP3 activation is commonly linked to signals generated by damaged mitochondria, including mtROS, oxidized mitochondrial DNA, altered mitochondrial membrane potential, and defective organelle turnover (Lackner et al., 2025). In this setting, mitophagy normally acts as a negative regulatory mechanism by removing damaged mitochondria before they accumulate sufficient danger signals to sustain inflammasome activation. Conversely, impaired PINK1/Parkin-dependent mitophagy may permit mitochondrial damage to persist and thereby favor NLRP3 priming or activation (Quinn et al., 2020). Evidence from neuroinflammatory disease models supports this general relationship: for example, NLRP3 deficiency has been reported to protect against intermittent hypoxia-induced neuroinflammation partly through enhancement of Parkin-dependent mitophagy, illustrating a reciprocal relationship between inflammasome activity and mitochondrial quality control (Wu et al., 2021). Although such evidence is not glaucoma-specific, it provides a biologically coherent framework for interpreting how reduced mitophagy may amplify inflammatory stress in the glaucomatous retina and optic nerve.

In glaucoma, this mechanism may involve both RGC-intrinsic and glia-mediated processes. In RGCs, insufficient mitophagy may worsen energy failure, oxidative stress, and susceptibility to apoptosis or other forms of regulated cell death (Zeng et al., 2020). In microglia and astrocytes, persistent mitochondrial injury may instead bias cells toward a pro-inflammatory phenotype, increasing cytokine release and lowering the threshold for inflammasome activation (Jassim et al., 2021b; Cullen and Sun, 2023). This distinction is important because the same mitochondrial defect may produce different consequences in different compartments: in neurons, it may primarily compromise survival capacity, whereas in glia, it may reshape the inflammatory environment in which neurons degenerate (Sun et al., 2018; Zhang et al., 2026). Recent reviews of mitophagy and neuroinflammation emphasize that PINK1/Parkin-dependent and receptor-mediated mitophagy are closely connected to inflammatory restraint, and that failure of mitochondrial clearance can propagate neuroinflammatory injury (Charmpilas et al., 2023). In the retina, mitophagy is increasingly viewed as a key component of mitochondrial homeostasis, with emerging relevance to RGC degeneration and glaucoma-related ocular neurodegeneration (Brooks et al., 2023; Skeie et al., 2021).

The mitophagy–NLRP3 axis may help explain why inflammation persists during glaucoma progression. Elevated IOP, ischemia, hypoxia, oxidative injury, or genetic stress can damage mitochondria (Venkatesan and Bernstein, 2025). When mitophagy is effective, these damaged mitochondria are removed and inflammatory signaling may subside. When mitophagy is impaired, however, damaged mitochondria continue to generate mtROS and other danger signals, sustaining inflammasome activation and glial reactivity. This may create a feed-forward cycle in which mitochondrial injury promotes inflammation, while inflammatory mediators further impair mitochondrial and lysosomal function (Kim et al., 2015; Mishra et al., 2021). Thus, early autophagic and mitophagic responses may be adaptive, whereas chronic disease may involve failed clearance and persistent inflammatory amplification.

Nevertheless, direct evidence that mitophagy failure activates NLRP3 in human glaucoma tissue remains limited. Current support comes mainly from glaucoma models showing mitochondrial dysfunction and neuroinflammation, together with broader neurodegeneration studies linking impaired mitophagy to inflammasome activation (Lu et al., 2025; Ahmed et al., 2026). Future studies should test whether restoring mitophagy can suppress NLRP3 activation, reduce IL-1β/IL-18 maturation, and preserve RGC survival. Such work would clarify whether mitophagy enhancement offers anti-inflammatory benefits in addition to mitochondrial protection.

## 6 Aging, NAD + signaling, and lysosomal–mitochondrial resilience

Aging provides an important biological context for interpreting dysfunction of autophagy, lysosomal competence, and selective mitophagy in glaucoma. With age, cells gradually lose mitochondrial efficiency, redox buffering capacity, proteostatic reserve, and lysosomal competence (Yan et al., 2026). These changes are particularly relevant to RGCs, which have high energy demands, long axons, and limited regenerative capacity (Visalli et al., 2026). In this setting, reduced nicotinamide adenine dinucleotide (NAD+) availability may act as a metabolic stress amplifier (Zhang et al., 2025). NAD+ is required for mitochondrial redox reactions and also serves as a substrate for sirtuins, a family of NAD + -dependent deacetylases that regulate mitochondrial metabolism, oxidative stress responses, DNA repair, autophagy, and mitophagy (Lagunas-Rangel, 2025). Reviews of glaucoma metabolism have therefore proposed NAD+/NADH redox imbalance as a contributor to RGC susceptibility and a potential biomarker or therapeutic target in glaucoma (Petriti et al., 2021).

Experimental and clinical observations support the translational importance of NAD + pathway. In glaucoma models, nicotinamide supplementation has been reported to protect RGCs and improve mitochondrial resilience, supporting the concept that metabolic reinforcement can increase neuronal stress tolerance (Williams et al., 2017). In humans, a crossover randomized clinical trial reported improved inner retinal function after nicotinamide supplementation in glaucoma patients, suggesting that NAD + augmentation may have measurable functional effects beyond IOP lowering (Hui et al., 2020). Additional clinical studies are now evaluating nicotinamide-based strategies for RGC function in glaucoma, reflecting growing interest in NAD + metabolism as a neuroprotective target (Efficacy of nicotinamide on retinal ganglion cell functions NCT06078605, 2025).

Nevertheless, NAD + supplementation should not be viewed as a simple autophagy activator. Its potential value may lie in restoring cellular resilience by improving mitochondrial metabolism, sirtuin activity, redox balance, and lysosomal–mitochondrial coupling. Future studies should determine whether NAD + augmentation enhances true mitophagic flux, preserves lysosomal competence, and benefits specific patient subgroups, particularly older individuals or fast progressors with evidence of mitochondrial vulnerability.

## 7 Glaucoma-associated genetic hits, selective autophagy, and mitochondrial quality control

Genetic forms of glaucoma provide an important entry point for linking disease susceptibility to autophagy-related quality-control pathways, including selective mitophagy. These genes do not converge on a single pathway, but several of them affect protein handling, selective autophagy, mitochondrial dynamics, or lysosomal stress. Among them, OPTN is the most direct bridge. Optineurin functions as a selective autophagy receptor and participates in ubiquitin-dependent cargo recognition (Sirohi and Swarup, 2016). Glaucoma-associated OPTN variants, including E50K and M98K, have been linked to altered vesicle trafficking, autophagy signaling, and retinal cell vulnerability (Swarup and Sayyad, 2018). Experimental work further shows that the M98K variant can induce autophagy-dependent retinal cell death through TBK1-mediated phosphorylation, suggesting that dysregulated selective autophagy may become harmful when it is excessive, misdirected, or uncoupled from normal cargo resolution (Swarup and Sayyad, 2018).

TBK1 provides a second genetic connection. Copy-number gains of TBK1 are associated with normal-tension glaucoma, and TBK1 is a key kinase that regulates selective autophagy by phosphorylating receptors such as OPTN. This makes the TBK1–OPTN axis particularly relevant to mitophagy and innate immune signaling. In neurodegeneration models, this axis has been proposed to coordinate mitochondrial clearance and inflammatory restraint, implying that TBK1 dysregulation in glaucoma may affect both RGC-intrinsic quality control and glia-mediated inflammatory responses (He et al., 2017).

By contrast, MYOC links autophagy to glaucoma through proteostatic stress in the anterior segment. Mutant myocilin is poorly secreted, accumulates in trabecular meshwork cells, and induces endoplasmic reticulum stress, proteasomal burden, and autophagy-related responses (Saccuzzo et al., 2023; Yan et al., 2024). Studies of myocilin processing indicate that normal myocilin turnover involves proteasomal and lysosomal pathways, whereas mutant or overexpressed myocilin can compromise proteasome function and induce autophagy. More directly, stimulation of autophagic flux using tat-beclin one peptide or torin two promoted mutant myocilin degradation and reduced elevated IOP in a MYOC-associated glaucoma mouse model. Thus, in MYOC-related disease, autophagy may be especially relevant to outflow-pathway proteostasis rather than to primary RGC mitophagy (Qiu et al., 2014; Kasetti et al., 2021).

OPA1 adds a mitochondrial-dynamics perspective. Although OPA1 is classically associated with dominant optic atrophy, its biology is highly relevant to glaucomatous RGC vulnerability because it regulates mitochondrial fusion, cristae integrity, and mitochondrial distribution (Ju et al., 2008). Experimental glaucoma studies indicate that OPA1 overexpression can protect RGCs, partly by promoting mitochondrial fusion and Parkin-mediated mitophagy. Conversely, impaired OPA1 function may favor mitochondrial fragmentation, axonal energy failure, and increased susceptibility to chronic stress (Hu et al., 2018).

Taken together, these genetic examples support a broader interpretation: glaucoma-associated genes may influence autophagy-related quality control at different biological levels, ranging from proteostatic stress and selective autophagy to mitochondrial dynamics and mitophagy. OPTN and TBK1 are closely connected to selective autophagy and mitophagy signaling; MYOC primarily imposes proteostatic and lysosomal burden in the trabecular meshwork; and OPA1 shapes mitochondrial dynamics and RGC resilience. This genetic diversity reinforces the need for a compartment-specific model. The same term, “autophagy dysfunction,” may refer to failed mutant-protein clearance in the outflow pathway, dysregulated selective autophagy in OPTN/TBK1-related disease, or impaired mitochondrial quality control in vulnerable RGCs. Future studies should therefore avoid treating genetic glaucoma as a uniform category and should instead define how each genetic lesion alters cargo recognition, lysosomal degradation, mitochondrial turnover, and cell-type-specific stress responses.

## 8 Molecular circuitry linking stress adaptation to neurodegeneration

If compartment-based analysis clarifies where autophagy remodeling occurs, the next question is how this remodeling is driven at the molecular level. Current evidence suggests that glaucomatous autophagy arises from the convergence of several interlocking pathways rather than from a single upstream trigger. These include energy sensing, selective cargo recognition, oxidative stress, mitochondrial surveillance, and broader proteostasis maintenance (Quinn et al., 2020; Jiménez-Loygorri et al., 2023; Zhang et al., 2026).

Among the best studied regulators are AMPK and mTORC1. Under normal conditions, AMPK promotes catabolic adaptation during energy stress, whereas mTORC1 suppresses autophagy in nutrient-replete states. In glaucoma-related contexts, however, these pathways appear less as simple switches and more as indicators of chronic metabolic disequilibrium. In human stem cell-derived OPTN (E50K) RGCs, impaired autophagic-lysosomal degradation has been observed together with AMPK activation and reduced mTORC1 signaling (Huang et al., 2024). At first glance, this might appear protective because AMPK activation and mTORC1 suppression are associated with autophagy induction. Yet in a chronic stress setting, the same pattern may instead signify a state in which autophagy is being signaled but cannot be effectively completed (Deus et al., 2020). The distinction is important. It suggests that pathway activation alone is not necessarily evidence of successful adaptation.

Optineurin (OPTN) is especially informative in this regard because it links inherited glaucoma susceptibility to selective autophagy machinery. Unlike many glaucoma-associated genes that influence risk more indirectly, OPTN is itself an autophagy receptor involved in ubiquitin-dependent cargo recognition and trafficking (Swarup and Sayyad, 2018; Wang et al., 2025). Human stem cell-derived RGCs carrying OPTN (E50K) display neurite retraction, altered excitability, apoptosis, and autophagy-related abnormalities (VanderWall et al., 2020). More recent work has shown impaired autophagic-lysosomal degradation in the same system, together with altered AMPK–mTORC1 signaling (Huang et al., 2024). Astrocytes carrying the same mutation also show autophagy dysfunction and can influence RGC phenotypes in co-culture (Gomes et al., 2022). These findings elevate OPTN from a gene of niche interest to a mechanistic bridge between glaucoma genetics and failed intracellular quality control.

Oxidative stress is another recurring driver. In the TM, redox imbalance has been linked directly to lysosomal dysfunction and impaired autophagic flux (Liton et al., 2008; Porter et al., 2013; 2015). In RGCs, ROS can initially induce autophagy under stress, but persistent oxidative burden also damages mitochondria, perturbs lysosomes, and increases the load of material that must be cleared (Zeng et al., 2020; Venkatesan et al., 2025). This can create a self-reinforcing loop in which damaged mitochondria generate more ROS while autophagic resolution becomes less efficient. This is one reason the move from bulk autophagy to mitophagy has been conceptually important.

The PINK1/Parkin pathway provides a central example. Under mitochondrial damage, PINK1 accumulates on the outer mitochondrial membrane and recruits Parkin, initiating selective mitochondrial clearance (Quinn et al., 2020). Several recent studies suggest that this pathway is relevant to glaucoma because mitochondrial dysfunction appears early in vulnerable RGCs, yet mitophagy is not always adequately engaged (Dai et al., 2018; Ju et al., 2023). Strengthening Parkin-mediated mitophagy can improve neuronal survival in experimental systems, which supports the idea that selective mitochondrial clearance is not a minor downstream event but a plausible vulnerability node (Zhuang et al., 2022; Hu et al., 2024).

At the same time, autophagy in glaucoma cannot be considered in isolation from the wider proteostasis network. Chaperones, the ubiquitin–proteasome system, lysosomes, and autophagy all interact (Shim et al., 2020; Osei Acheampong and Insolera, 2026; Wang J. et al., 2026). Disturbance in one arm can increase pressure on the others. Chronic oxidative stress, mitochondrial injury, aging-related lysosomal decline, and endoplasmic reticulum stress may therefore converge in a broader collapse of quality-control integration (Venkatesan and Bernstein, 2025). In this context, increased autophagosome abundance may reflect compensation, incomplete degradation, or unresolved cargo burden rather than improved cellular state.

Taken together, these pathways suggest that glaucoma progression may involve a shift from adaptive stress signaling to pathological lock-in. Early stress can still be met by coordinated changes in AMPK, mTORC1, autophagy, and mitophagy. With persistent injury, that coordination may be lost: cargo recognition becomes inefficient, lysosomal processing falters, damaged mitochondria accumulate, and stress signaling persists without resolution (Porter et al., 2013; Dai et al., 2018; Quinn et al., 2020; Zhuang et al., 2022; Jiménez-Loygorri et al., 2023; Chaphalkar et al., 2024; Huang et al., 2024; Hu et al., 2024). The therapeutic implication is straightforward but important. The goal is unlikely to be indiscriminate pathway activation. It is to restore functional coupling among stress sensing, cargo recognition, lysosomal degradation, and mitochondrial turnover.

## 9 Therapeutic implications, biomarkers, and translational challenges

The therapeutic appeal of targeting autophagy-related quality-control pathways lies in their position at the intersection of several pathogenic processes relevant to glaucoma. Elevated IOP, oxidative stress, mitochondrial dysfunction, proteostatic decline, and inflammatory signaling all converge, directly or indirectly, on autophagic machinery (Porter et al., 2015; Jassim and Inman, 2019; Jiménez-Loygorri et al., 2023). In principle, this gives autophagy-centered interventions a broader reach than single-target neuroprotective strategies. At the same time, this breadth explains why translation has been slow. A pathway that is adaptive in one context may be insufficient or maladaptive in another.

Several experimental interventions support the idea that autophagy modulation can be beneficial. Rapamycin and fasting-related paradigms can sustain autophagy responses and promote RGC survival after retinal ischemia/reperfusion injury (Russo et al., 2018). Other studies suggest that antioxidants such as N-acetylcysteine may influence autophagy-related pathways while reducing oxidative burden in selected glaucoma models (Yang et al., 2012; Sano et al., 2019). These observations indicate that stress adaptation can, under some conditions, be therapeutically reinforced.

However, therapeutic prioritization should not be based simply on whether a compound increases or decreases autophagy markers. A more mechanistically guided framework should first define the dominant biological context. In early or acute stress, when lysosomal degradation remains relatively competent, transient enhancement of autophagy or mitophagy may help remove damaged proteins and organelles. In chronic disease with partial lysosomal impairment, strategies that combine mitochondrial cargo recognition with lysosomal support may be more rational than upstream pathway activation alone. In advanced disease, where lysosomal failure, axonal degeneration, and glial inflammatory amplification may already be established, broad autophagy induction is unlikely to be sufficient and may need to be replaced or supplemented by approaches aimed at lysosomal restoration, mitochondrial stress reduction, anti-inflammatory modulation, and axonal support. Thus, the therapeutic question is not whether autophagy should be activated, but which component of the quality-control pathway is limiting in a given compartment and disease stage.

Representative interventions are summarized in Table 2 according to this context-dependent logic. The table is organized not only by intervention class, but also by the presumed limiting defect, including upstream stress signaling, lysosomal competence, mitochondrial cargo recognition, proteasomal mitochondrial quality control, oxidative stress, glial inflammatory amplification, and compartment-directed delivery. This framework is intended to move the translational discussion from a descriptive list of interventions toward a prioritization scheme based on disease stage, ocular compartment, and pathway bottleneck.

**TABLE 2:** Context-dependent therapeutic prioritization of autophagy- and mitochondrial quality-control strategies in glaucoma.

Biological context	Presumed limiting defect	Preferred intervention logic	Representative strategy	Expected benefit	Key caveat
Acute or early retinal stress	Autophagy/mitophagy inducible; lysosomes relatively competent	Support adaptive autophagic flux	Rapamycin, fasting-related paradigms	Improve stress clearance and RGC survival	May not apply to chronic glaucoma
Chronic RGC mitochondrial stress with partial lysosomal competence	Damaged mitochondria recognized inefficiently	Enhance selective mitochondrial tagging and clearance	PR-619, S3, SHMT2-related modulation	Improve mitochondrial quality control	Requires downstream lysosomal capacity
Chronic disease with lysosomal impairment	Degradation, acidification, or fusion is limiting	Restore lysosomal competence before or with mitophagy enhancement	TFEB/lysosomal biogenesis approaches; lysosomal acidification support	Improve completion of autophagic/mitophagic flux	Specific glaucoma evidence still limited
Severe mitochondrial damage with incomplete lysosomal clearance	Mitochondrial danger signals persist	Combine mitochondrial stress reduction with alternative quality-control pathways	Antioxidants, proteostasis support, proteasomal mitochondrial protein turnover	Reduce mtROS and damaged mitochondrial protein burden	Proteasomal MQC remains underexplored in glaucoma
Conventional outflow pathway dysfunction	ECM remodeling, cytoskeletal stress, senescence, lysosomal stress	Prioritize lysosomal proteostasis and mechanoadaptation	Autophagic flux restoration in TM/Schlemm’s canal cells	Improve outflow tissue resilience	Mitophagy may not be the dominant target
Glial inflammatory amplification	Mitochondrial stress and inflammasome activation in glia	Combine mitophagy support with anti-inflammatory modulation	NLRP3/caspase-1 inhibition; mitochondrial stress reduction	Reduce inflammatory amplification around RGCs	Cell-type specificity remains difficult
Patient-level translation	Unknown dominant biological vulnerability	Use biomarkers to guide selection	OCT/OCTA, flavoprotein fluorescence, aqueous humor biomarkers	Identify fast progressors or mitochondrial-vulnerable patients	No validated mitophagy biomarker yet
MYOC-related or ER-stress-dominant outflow dysfunction	Misfolded/aggregated myocilin and ER proteostatic burden	Enhance aggrephagy/ER-autophagy-related clearance and reduce ER stress	ER–autophagy modulation; autophagic flux enhancement; chemical chaperone or proteostasis support	Improve mutant protein clearance and TM cell survival	Most evidence comes from MYOC models; generalizability to non-MYOC POAG remains uncertain
Oxidative stress-dominant TM dysfunction	Lysosomal damage, basification, and impaired flux	Support lysosomal quality control and possible lysophagy-related recovery	Lysosomal acidification support; TFEB/lysosomal biogenesis strategies	Restore degradative capacity and outflow tissue resilience	Direct lysophagy evidence in glaucoma remains limited
Mechanically stressed outflow pathway	Cytoskeletal remodeling, focal adhesion turnover, ECM imbalance	Restore autophagy-linked mechanoadaptation	Autophagic flux restoration in TM/Schlemm’s canal cells	Reduce stiffness-associated outflow resistance	Need better compartment-specific biomarkers
RGC/optic nerve degeneration	Damaged mitochondria and axonal energy failure	Enhance mitophagy if lysosomal competence is preserved or restorable	PR-619, S3, SHMT2/PINK1-Parkin modulation	Improve mitochondrial quality control and RGC resilience	Requires downstream lysosomal degradation

This table organizes potential intervention strategies according to the presumed limiting biological defect rather than by compound class alone. In early or acute stress, autophagy or mitophagy enhancement may be beneficial if lysosomal degradation remains competent. In chronic disease with partial lysosomal impairment, selective mitochondrial cargo recognition may need to be paired with lysosomal support. In advanced disease, where lysosomal failure, mitochondrial danger signaling, and glial inflammation may coexist, broader strategies targeting lysosomal competence, mitochondrial stress reduction, proteostasis, and inflammatory amplification may be required.

Still, broad autophagy induction is unlikely to be the whole answer. If lysosomal degradation is already compromised, further pathway activation may increase autophagosome or mitophagosome burden without restoring effective clearance (Porter et al., 2013; Lee et al., 2021; Dixon et al., 2023). Mitophagy-oriented therapy should therefore not be viewed as bypassing the need for lysosomal competence. Its potential benefit depends on the biological context. When lysosomal capacity is preserved or only partially impaired, enhancing upstream mitochondrial recognition, Parkin recruitment, or mitophagosome formation may improve the selectivity and efficiency of damaged mitochondrial clearance. Under these conditions, interventions such as PR-619, S3, or SHMT2-related modulation may be beneficial because the downstream degradative system remains capable of completing mitophagic flux (Dai et al., 2018; Zhuang et al., 2022; Hu et al., 2024).

By contrast, when lysosomal degradation is severely impaired, upstream mitophagy initiation alone is unlikely to be sufficient. In such settings, therapeutic strategies would need to restore lysosomal acidification, hydrolase activity, autophagosome–lysosome fusion, or lysosomal biogenesis before mitophagy enhancement can produce durable mitochondrial clearance. This distinction is important for glaucoma because lysosomal dysfunction may be partial and reversible in some stressed trabecular meshwork cells or early RGC injury states, but more advanced in aged or chronically injured tissues. Therefore, mitophagy enhancement should be prioritized mainly when lysosomal function is still competent or restorable, whereas combined lysosomal–mitochondrial repair may be required in chronic disease.

In addition, mitochondrial quality control is not exclusively lysosome dependent. Before complete organelle removal through mitophagy, damaged mitochondrial proteins can also be handled through ubiquitin–proteasome-dependent mechanisms, including extraction and degradation of outer mitochondrial membrane proteins. These processes may reduce mitochondrial danger signaling, limit accumulation of damaged mitochondrial components, and provide a partial protective mechanism when lysosomal degradation is delayed or incomplete. Although such proteasomal mitochondrial quality-control pathways remain insufficiently studied in glaucoma, they are conceptually relevant because they suggest that therapeutic mitochondrial repair should not be restricted to canonical mitophagic flux alone. Future glaucoma studies should therefore distinguish among mitochondrial tagging, proteasomal removal of damaged mitochondrial proteins, mitophagosome formation, lysosomal degradation, and restoration of mitochondrial function.

A further translational issue is cellular specificity. Autophagy is required not only in RGCs, but also in photoreceptors, RPE, Müller glia, and trabecular meshwork cells. Thus, non-selective autophagy activation may have unintended effects across the eye. Future strategies should therefore consider local ocular delivery, dose and timing optimization, compartment-aware formulations, and, for gene-based approaches, vector or promoter selection that enriches expression in the intended cell population. Biomarker-guided patient selection will also be important, because mitophagy-enhancing therapy is most likely to benefit patients whose disease is driven by mitochondrial vulnerability or rapid neurodegenerative progression. Overall, RGC-selective mitophagy repair may be more attractive than global autophagy modulation, but achieving sufficient cellular and temporal precision remains technically challenging.

Another key implication of the compartment-based framework is that therapeutically relevant defects may differ between tissues. In the anterior segment, the priority may be restoration of lysosomal competence, proteostasis, and mechanoadaptation in TM and Schlemm’s canal cells (Porter et al., 2013; Liton, 2016; Shim and Liton, 2022). In the posterior segment, the main target may be preservation of RGC proteostasis, mitochondrial turnover, and axonal integrity (Russo et al., 2018; Lee et al., 2021; Dixon et al., 2023). Interventions that help one compartment may not automatically benefit another. This becomes even more important when glial dysfunction is considered. Autophagy-related abnormalities in OPTN (E50K) astrocytes suggest that durable neuroprotection may require more than direct neuronal rescue (Gomes et al., 2022).

Despite these mechanistic advances, the field still lacks robust biomarkers. At present, there is no validated way to monitor autophagic flux or mitophagy competence directly in living glaucoma patients. Clinical assessment relies on structural and functional endpoints such as OCT, retinal nerve fiber layer thickness, ganglion cell complex measurements, and visual field progression (Poli et al., 2018; Sayah et al., 2020; Hohberger et al., 2021; Strat and Ganapathy, 2025). These remain essential, but they are downstream measures. They do not identify whether a patient’s dominant vulnerability lies in mitochondrial quality control, lysosomal insufficiency, or another aspect of stress biology. Future progress will likely require multimodal biomarker strategies that combine imaging, fluid-based measurements, and metabolic or mitochondrial indicators.

Imaging biomarkers may also help stratify patients for mechanism-based therapy. OCT and OCT-angiography already provide clinically useful measures of retinal nerve fiber layer thinning, ganglion cell complex loss, optic nerve head remodeling, and retinal or peripapillary microvascular compromise; OCTA studies have consistently reported reduced peripapillary and macular vessel density in glaucomatous eyes, although these vascular parameters are best interpreted together with structural OCT rather than as stand-alone indicators (WuDunn et al., 2021; Hong et al., 2024). These parameters do not directly report autophagic flux or mitophagy competence, but they can identify structural progression and vascular stress. More mechanism-oriented approaches may add metabolic information. Retinal flavoprotein fluorescence has been proposed as a non-invasive indicator of mitochondrial oxidative stress in retinal disease and has been reported to be elevated in primary open-angle glaucoma, suggesting potential value for detecting early retinal metabolic stress (Field et al., 2011; Zhou et al., 2022). Mitochondrial PET tracers, such as 18F-BCPP-EF for mitochondrial complex I, remain largely exploratory in ophthalmology, but studies in neurodegenerative disease models and human brain imaging indicate that they can quantify mitochondrial complex I-related bioenergetic abnormalities *in vivo* (Tsukada et al., 2016). In future studies, combining OCT/OCT-A progression metrics with mitochondrial or metabolic imaging and aqueous humor biomarkers, including extracellular vesicles, cytokines, oxidative-stress proteins, or metabolomic signatures, may help identify fast progressors or patients with mitochondrial vulnerability who are most likely to benefit from mitophagy-targeted therapies (Wang and Wei, 2023; Shin et al., 2024).

This difficulty is compounded by a familiar methodological problem: static markers do not reliably distinguish pathway activation from pathway competence. LC3 and p62 signals may reflect productive autophagy, stalled flux, or accumulated burden (Porter et al., 2013; Zeng et al., 2020; Lee et al., 2021). Mitochondrial injury markers do not by themselves prove productive mitophagy (Klionsky et al., 2021; Jiménez-Loygorri et al., 2023). This ambiguity continues to limit comparability across studies and complicates interpretation of therapeutic experiments.

There are also broader translational barriers. Glaucoma is biologically heterogeneous (Sayah et al., 2020; Giorgina and Emmanuelle, 2024). Acute injury models, chronic ocular hypertension models, and genetic stem cell-based systems each capture different slices of the disease (Russo et al., 2018; Lee et al., 2021; Gomes et al., 2022; Dixon et al., 2023). Drug delivery and treatment timing remain unresolved. A therapy that is useful during early compensation may have little value once structural degeneration is established. Clinical trials are difficult because progression is often slow and mechanism-based endpoints remain underdeveloped (Dervisevic et al., 2016; Sayah et al., 2020).

**FIGURE 3:**
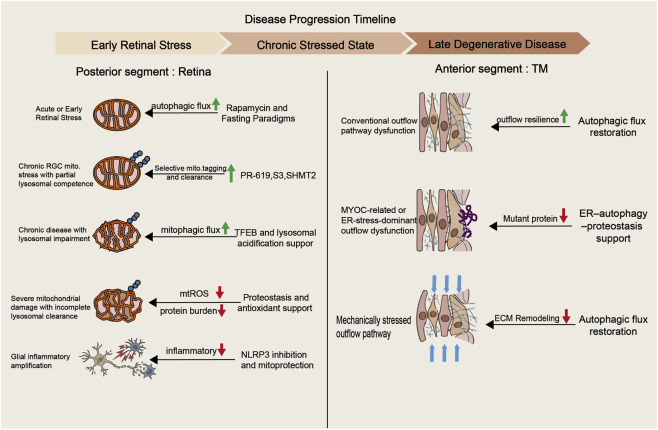
Therapeutic opportunities in targeting autophagy, lysosomal competence, and selective mitophagy in glaucoma. Potential interventions can be conceptually organized across several levels, including reduction of chronic biomechanical stress through intraocular pressure lowering, restoration of autophagy-related signaling and lysosomal competence, and selective enhancement of mitochondrial quality control. In posterior segment, focuses on selective mitophagy and mitochondrial quality control (e.g., PINK1/Parkin enhancement, PR-619) to preserve RGC survival, given their metabolic dependence on mitochondrial integrity. In anterior segment, emphasizes restoring autophagic flux to mitigate mechanical stress, ECM remodeling, and mutant protein burden (e.g., MYOC-related ER stress), thereby improving conventional outflow facility and lowering IOP.

Even so, the overall direction is encouraging. The most plausible path forward is not to abandon autophagy-centered therapy, but to refine it. Glaucoma will likely need to be stratified more explicitly by dominant stress biology rather than by IOP alone. Biomarker development should focus on flux competence and selective mitochondrial quality control rather than on single static markers. Therapeutic strategies may ultimately need to combine IOP lowering with compartment-prioritized restoration of intracellular quality control (Quinn et al., 2020; Zhuang et al., 2022; Chaphalkar et al., 2024). A stage-aware and compartment-aware framework for therapeutic targeting of autophagy, lysosomal competence, and selective mitochondrial quality control is illustrated in Figure 3.

## 10 Conclusions and future directions

The evidence reviewed here supports a conceptual shift in how glaucoma is interpreted. It is increasingly difficult to regard the disease as a simple pressure-driven optic neuropathy. A more accurate view is that glaucoma is a chronic, multicompartment neurodegenerative disorder in which biomechanical stress, oxidative injury, mitochondrial dysfunction, and declining proteostatic capacity interact over time. Within this broader framework, autophagy and its selective mitochondrial branch, mitophagy, are not peripheral phenomena. They are central regulators of whether ocular tissues can adapt to stress, preserve homeostasis, and resist degeneration.

A central conclusion of this review is that the role of autophagy in glaucoma cannot be reduced to a simple beneficial-versus-harmful binary. The available literature instead supports a stage-dependent and compartment-specific model. In the conventional outflow pathway, autophagy appears to contribute to mechanoadaptation and maintenance of aqueous humor homeostasis, but chronic oxidative and biomechanical stress may impair lysosomal function and promote outflow failure. In RGCs, autophagy may initially support survival during acute or early injury, yet become insufficient or dysregulated as stress persists. In the optic nerve and glial microenvironment, further vulnerabilities arise from axonal transport demands, altered trophic support, and non-cell-autonomous stress amplification. The common thread is not simply altered autophagy level, but a progressive decline in effective quality control across interconnected compartments.

Within this model, mitophagy deserves particular emphasis. This is not because bulk autophagy has become unimportant, but because selective mitochondrial turnover appears especially relevant to the biology of vulnerable RGCs. The emerging literature on PINK1/Parkin signaling, optineurin-linked autophagic dysfunction, and mitophagy-enhancing rescue strategies suggests that mitochondrial quality control may be one of the more tractable mechanistic entry points for future therapy. Even so, the field should remain cautious. Current evidence is promising but still fragmented across model systems, and the biological meaning of many autophagy-related markers remains context dependent.

Several priorities follow from this. First, future studies need better temporal resolution. We still do not know precisely when adaptive autophagy shifts toward chronic insufficiency, whether this transition differs among ocular compartments, or how it relates to structural progression. Second, glaucoma research would benefit from a more explicit compartment-based design, asking not only whether autophagy is altered but where the dominant defect lies. Third, clinically useful biomarkers of autophagy and mitophagy competence remain urgently needed. Without them, mechanism-based therapy will remain difficult to target and evaluate. Fourth, therapeutic development should focus less on indiscriminate pathway modulation and more on restoring functional coupling among stress sensing, cargo recognition, lysosomal degradation, and selective mitochondrial turnover.

More broadly, glaucoma should be situated within the wider biology of age-related neurodegeneration. Many of the themes discussed here—mitochondrial dysfunction, proteostatic fragility, selective autophagy defects, and maladaptive glial responses—resonate with work in other chronic neurodegenerative disorders. At the same time, glaucoma offers a distinct advantage as a model system: it links anterior-segment stress and posterior-segment neurodegeneration within a single organ and allows structural progression to be measured with unusual precision. For that reason, studying autophagy in glaucoma may not only improve glaucoma therapeutics but also sharpen broader ideas about how compartmentalized failure of cellular quality control drives chronic neuronal vulnerability.

Future therapeutic development should therefore be guided by the dominant pathway bottleneck rather than by autophagy activation alone. If lysosomal degradation is preserved, selective mitophagy enhancement may improve mitochondrial clearance. If lysosomal function is compromised, mitophagy-oriented strategies will need to be combined with lysosomal restoration or alternative mitochondrial quality-control mechanisms. This context-dependent framework may provide a more realistic route for translating autophagy biology into glaucoma therapy. In practical terms, the most promising future direction is not simply to activate autophagy more aggressively. It is to determine which component of the quality-control system fails, in which compartment, and at which stage of disease, and then to restore that function as selectively as possible. That is a more modest conclusion than some of the early enthusiasm in the field might have suggested, but it is also a more durable (Giorgina and Emmanuelle, 2024; Efficacy of nicotinamide on retinal ganglion cell functions NCT06078605, 2025) one.
